# Effects of Elective Caesarean Sections in Healthy Near-Term Ewes on Subsequent Reproductive Performance

**DOI:** 10.3390/ani14060925

**Published:** 2024-03-17

**Authors:** Katja Voigt, Mara Theisges, Yury Zablotski, Frank Weber, Holm Zerbe

**Affiliations:** Clinic for Ruminants with Ambulatory and Herd Health Services, Centre for Clinical Veterinary Medicine, Ludwig-Maximilians-Universität München, Sonnenstr. 16, 85764 Oberschleissheim, Germany; m.theisges@med.vetmed.uni-muenchen.de (M.T.); y.zablotski@med.vetmed.uni-muenchen.de (Y.Z.); weber@lmu.de (F.W.); h.zerbe@lmu.de (H.Z.)

**Keywords:** caesarean section, fertility, litter size, dystocia, sheep

## Abstract

**Simple Summary:**

In cases of birth difficulties in sheep, caesarean sections can be life-saving. It is, however, important for farmers to know whether the ewe is likely to conceive again. In field cases following birth difficulties, many factors such as the problematic delivery itself or any underlying health problems will also influence these outcomes. In order to assess a potential direct effect of surgery, it is important to study healthy animals undergoing pre-planned operations. Animals from a university sheep flock were, therefore, studied retrospectively by evaluating their breeding records. Caesarean sections were performed by veterinary students as part of their training, and the subsequent breeding outcomes were compared to animals which had only lambed naturally. There was no negative effect of caesarean surgery on the subsequent pregnancy rate or on lamb viability or birth weights. However, in the pregnancy immediately following a surgical delivery, a smaller number of lambs was born per ewe, and a higher number of mating attempts was necessary to achieve pregnancy. The number of lambs born returned to pre-caesarean levels in further subsequent pregnancies. We, therefore, conclude that caesarean sections in sheep have very little influence on their long-term reproductive performance; this procedure is, therefore, worthwhile.

**Abstract:**

Post-surgical reproductive performance following ovine caesarean section has not been well studied. To assess any direct effects of surgical delivery in the absence of confounders such as dystocia or underlying diseases, we studied elective surgery performed in healthy animals for teaching purposes. Four hundred and eleven paired breeding records following vaginal delivery (*n* = 233), elective caesarean section (*n* = 122), and subsequent further vaginal deliveries in animals with a history of one prior elective caesarean operation (*n* = 56) were evaluated retrospectively. The overall subsequent pregnancy rate was 95%. Multivariable statistical analyses did not reveal any significant influence of planned caesarean surgery on subsequent conception, stillbirth, perinatal lamb mortality, lamb birth weights, or the incidence of premature foetal death (mummification and abortion). A significantly higher number of mating attempts was, however, necessary. Also, a significant reduction in litter size was seen in the first pregnancy immediately following a surgical delivery in comparison to animals which had previously only delivered vaginally (*p* = 0.001), but litter size returned to pre-caesarean levels in further follow-up pregnancies in animals with a history of one elective caesarean section (*p* = 0.436). Subsequent long-term reproductive performance of sheep following elective caesarean section is thus excellent, and the results encourage retention for breeding.

## 1. Introduction

Caesarean section is a potentially life-saving procedure in small ruminant obstetrics. Reported cumulative ewe survival rates until hospital discharge or until day 7 post surgery are generally satisfactory in emergency situations following dystocia, ranging from 81% to 95%, with no obvious differences between hospitalized and field cases [1,2,3,4,5,6]. In addition to any possible direct effects of the procedure itself, dam survival and any potential consequences for subsequent fertility and fecundity are, however, also influenced by the concurrent presence of dystocia or underlying health conditions [5]. It is, therefore, difficult to assess the influence of surgery alone on short- and long-term outcomes of ovine caesarean section in any field cases [7].

A negative effect of caesarean surgery on subsequent fertility is assumed in domestic animal species such as, for instance, cattle, as well as in humans, but it is difficult to quantify amongst the variety of potentially influential factors, particularly if performed following dystocia. A systematic review and meta-analysis of the subsequent fertility of women following Caesarean section revealed an average reduction in fertility by close to 10% in comparison to vaginal delivery in the examined publications, but the authors highlight methodical weaknesses of many of the examined studies, which may have led to significance without a true causal connection. Notably, the two reviewed studies focusing on elective caesarean sections due to foetal breech presentation (thus excluding any potential influence of dystocia) did not identify a significant effect, and the authors, therefore, suggest that future focus on this matter should be put on elective procedures to exclude any potentially confounding effects [8].

Subsequent fertility and pregnancy outcomes following ovine dystocia-related caesarean section have not been extensively studied, and publications reporting post-surgical fertility in sheep are often hampered by low case numbers due to high culling rates, owners deciding against re-breeding, or loss of animals to detailed follow-up. Under these circumstances, post-surgical conception rates of between 90% and 100% have been reported [1,3,5,9,10]. While these are overall very satisfactory figures, particularly in comparison to cattle, where reported post-surgical conception rates in re-bred animals range between 48.6% and 75% [11,12,13,14], a selection bias cannot be ruled out in these clinical case cohort studies, as only an owner-selected proportion of the operated animals were re-bred. The frequent lack of a control group following natural delivery further limits these results. Studies examining the future reproductive performance of ewes following elective caesarean surgery in clinically healthy animals are, therefore, highly desirable to better understand the potential impact of the procedure itself and to exclude any potential confounding effects of dystocia or other health conditions. Only three such reports have, however, been published to date: one refers to 17 New Zealand Romney ewes operated around term for scientific purposes in comparison to a control group of the same size following vaginal delivery. The authors did not observe any differences in subsequent lambing percentage (88.2% in both groups) or the number of lambs born [15]. Another publication refers to subsequent breeding outcomes following surgical removal of the foetuses of 32 ewes of various native Egyptian breeds for scientific purposes at different stages of gestation. All ewes subsequently conceived. The number of lambs born to these ewes in the pregnancy following the operation was slightly higher than before the procedure. No control group was, however, included [16]. The most detailed study on the subject was conducted in Norway on Texel, Dala, and crossbred ewes, initially including 162 ewes operated on near term for teaching purposes in comparison to the same number of control animals following vaginal delivery. Of the initial animals, 113 (69.8%) and 116 (71.6%), respectively, were subsequently re-mated. There was no significant difference in subsequent pregnancy rates in the re-mated animals (89.4% in the operated as opposed to 89.7% in the control group), but ewes from the surgical group showed a lower number of triplet births and thus gave birth to a significantly lower number of lambs in the following season. They also had a significantly higher percentage of stillborn lambs, together leading to a significantly reduced number of live-born lambs per ewe. No significant differences in lamb birth weights, neonatal losses, or lamb weight gain were observed by these authors [7]. While the two groups were checked for statistically significant differences in potentially confounding factors prior to group comparison, multivariable models were only applied for the examination of lamb birth weight and weight gain. Potential influences other than the mode of previous delivery, such as, for instance, age and parity on pregnancy rate, litter size, and stillbirth rate were thus not directly assessed in this study.

Data on the future reproductive performance of healthy sheep undergoing elective near-term surgical delivery thus remain sparse and, in part, contradictory, but are crucial for understanding potential direct impacts of caesarean surgery on future reproduction. We, therefore, retrospectively analysed clinical and flock management data of ewes undergoing elective caesarean sections carried out for teaching purposes in comparison to vaginal deliveries, applying multivariable analyses to account for the potentially confounding influence of further parameters such as, for instance, parity, age, litter size, and management protocols, to contribute to a better understanding of a possible direct influence of surgical delivery (in the absence of dystocia) on the future reproductive performance of ewes. In addition to assessing the immediate post-surgical pregnancy, we were also able to analyse further subsequent parturitions in order to evaluate potential true long-term effects of caesarean section.

## 2. Materials and Methods

A retrospective whole-flock study was performed to assess parturition records of all sheep that lambed between 1 January 2009 and 30 June 2023 in an approximately 60-ewe flock of Bavarian Alpine Sheep (Bayerisches Bergschaf) kept for educational purposes at the agricultural facilities of a veterinary university hospital. The history of the immediately preceding parturition (falling into 2008) was also taken into account for any animals lambing in 2009. A total of 636 parturition records from 257 sheep were available for initial evaluation within the studied period. These included 302 vaginal deliveries without any prior caesarean section, 156 first and 80 second caesarean deliveries, 85 natural parturitions following a first caesarean section, and 13 vaginal deliveries following a second operation. All caesarean sections were elective in nature except for two occasions. One unplanned caesarean section due to ringwomb and one salvage caesarean section due to illness of the dam were performed during the study period. Both animals were not re-bred after these events and thus did not confound assessment of subsequent breeding outcomes for the elective procedure. The utilised breed has good potential for out-of-season breeding, but still shows some seasonal variation in fertility [17]. The animals were kept on deep straw bedding with seasonal access to an outdoor pasture area. The flock was of a good health status and free from infectious abortions, and details of flock health management have been previously published [18]. Detailed flock and individual records were kept continuously and were evaluated retrospectively. Data on placental shedding were, however, only available for sheep lambing between 2009 and 2023, since these details had not been recorded for parturitions falling into 2008.

The overall reproductive aim of the flock was the provision of pregnant animals for teaching purposes throughout the year according to the students’ curriculum. An average of 43 parturitions per year thus took place in small groups of sheep up to eight times throughout a year, with 33.1% falling into spring, 25.8% into autumn, 24.5% into summer, and 16.6% into winter. Oestrus synchronization of up to 10 sheep at a time was performed using either intravaginal sponges containing 20 mg flugestone acetate (Chronogest^®^ CR, Intervet, UK) for a duration of 14 days or a protocol of two intra-muscular injections of 0.15 mg cloprostenol (Dalmazin SYNCH, Selectavet, Germany) 9 to 10 days apart. Depending on the availability of the product(s), 300 to 500 IU equine chorionic gonadotropin (eCG) (Intergonan^®^, Intervet, Germany or Pregmagon^®^, Ceva, Germany) were injected intra-muscularly at the time of sponge removal/second injection of cloprostenol in some but not all occasions. Healthy, fertile rams were introduced the day after sponge removal/second injection of cloprostenol for a three-day mating period in groups of a maximum of three ewes per ram. For the purposes of calculating days of gestation, the second day spent with the ram (48–72 h after completion of the synchronization protocol) was defined as the mating day for all synchronized animals. Animal groups not intended for caesarean section were mated without any prior synchronization for a short period of 2–3 weeks to achieve a narrow lambing time for management reasons, thus sacrificing maximum pregnancy rates. Pregnancy was confirmed with transabdominal ultrasonography using a 5 MHz convex probe at approximately days 35 and 50 of gestation (Honda HS 101 V, Physia GmbH, Neu-Isenburg, Germany). Ewes confirmed as not pregnant were retained in the flock and again included in later synchronization/mating groups. All ewes were allowed a minimum of three mating rounds before they were classified as infertile (and eventually removed from the flock for that reason) due to the expected low immediate pregnancy rates following the above reproductive protocols and frequent out-of-season breeding. In animals not falling pregnant at the first mating attempt, assessment of conception was thus based on the outcome of a series of these permitted mating rounds, with the individual attempts often taking place in different seasons and sometimes involving different protocols in one animal. A minimum period of 2 months (median: 5 months) passed between any repeated mating attempts. Ewes that did not conceive during the first mating attempt were thus often re-mated at different times of year. The median rest period (time between parturition and first mating attempt) was 260 days (approximately 8.5 months), and there was no significant difference in the rest period between the three studied parturition groups. The median time between two parturitions was 477 days (1.3 years) in the studied flock.

For statistical analyses, the reproductive management programmes were categorized as no synchronization (none), synchronization with the use of eCG, and synchronization without the use of eCG.

Parturition was expected at around day 150 of pregnancy [18]. The animals were transferred to the hospital facilities for close round-the-clock monitoring approximately one week before their calculated lambing date. Following parturition, they remained there for several days before returning to the agricultural premises. In case of delays in the birth process, timely assistance at lambing was provided. Any weak lambs were cared for intensively and given colostrum or assistance with suckling if required. Details of these standard procedures and flock health management have been previously published [18].

Elective near-term caesarean sections were performed by groups of final year veterinary students as part of their clinical training on day 147, 148, or 149 of gestation under close supervision by an experienced veterinary surgeon [18]. All procedures complied with EU Directive 2010/63/EU and German animal welfare legislation, and were approved by the Upper Bavarian District Government under licenses no 55.2-1-54-2531.3-01-06, 55.2-1-54-2532.3-23-09, 55.2.1-54-2532.3-38-13, ROB-55.2Vet-2532.Vet_03-17-92 and ROB-55.2-2532.Vet_02-23-56. Until 2022, a maximum of two caesarean sections were allowed in a ewe’s lifetime under these licenses, while the number of permitted operations per ewe was reduced to one from 2023 onwards.

Different students were involved in the different operations. The students had previously received surgical training using slaughterhouse organs, suture pads, and by assisting in surgical procedures. To support foetal lung maturation, 2 mg dexamethasone were applied subcutaneously to all animals designated for surgery 36 h before the planned operation. This was followed by 10 mg dexamethasone subcutaneously 12 h before the procedure [19]. If possible, ewes that had lambed naturally at least once were preferentially selected for elective caesarean section to optimize their mothering capabilities. All unsynchronized animals and any surplus synchronized sheep lambed naturally.

All caesarean sections were carried out according to a standard protocol following a left flank incision in right lateral recumbency under local anaesthesia with procaine hydrochloride (various suppliers) as previously described [18]; in case of multiple lambs, both uterine horns were incised. The lambs were presented to the ewes immediately after removal from the uterus to encourage the ewe–lamb bonding process. The ewes were subsequently sedated using 0.1 mg/kg body weight (bw) xylazine intramuscularly (various suppliers) because of the prolonged suture time due to inexperienced surgeons (veterinary students). To counter-act uterine contractions induced by the side-effects of xylazine, 0.15 mg clenbuterol (Planipart, Boehringer Ingelheim, Ingelheim am Rhein, Germany) were applied intravenously per animal as approved by the experimental licenses. The uterine incisions were closed using a double-layer continuous inverting pattern (Cushing suture, Surgicryl monofilament, USP 1–EP 4, SMI AG, Saint Vith, Belgium). The abdominal cavity was closed in four layers as previously described in detail [18]: (1) joint suture of peritoneum and transverse muscle through simple continuous suture, (2) joint closure of the two oblique abdominal muscles using simple continuous suture, (3) subcutaneous adaptation, and (4) skin closure using metal staples (Manipler AZ–35W, B Braun, Melsungen, Germany). A peri-operative course of at least five days of amoxicillin was applied in all cases (15 mg/kg body bw, various suppliers), and at least three days of non-steroidal anti-inflammatory drugs were administered peri-operatively in each animal, using either meloxicam (various suppliers, 0.5 mg/kg bw subcutaneously) or flunixin-meglumine (various suppliers, 2.2 mg/kg bw subcutaneously). From 2018 onwards, additional application of metamizole (20 mg/kg bw intravenously, various suppliers) was required by the experimental license. Immediately after completion of surgery, 70 µg carbetocin (Depotocin, Veyx, Schwarzenborn, Germany) were applied subcutaneously per animal [18] to aid uterine contraction and placental shedding.

A previous parturition was a prerequisite for inclusion in the study. Once animals had completed two caesarean sections, they were usually removed from the flock. A small number of ewes went on to have further pregnancies following a second caesarean section. Any follow-up pregnancies to these second caesarean sections were excluded from the analyses due to low case numbers, and to avoid any potential cumulative effects of two surgical deliveries.

From the available data, reproductive performance was measured as subsequent conception (based on the cumulative outcome of permitted mating attempts), the number of synchronization/mating attempts leading to this pregnancy, the subsequent litter size, number of liveborn lambs, stillbirth, and perinatal mortality rates, lamb birth weights, and the incidence of premature foetal death during subsequent pregnancy (mummification and abortion). To take into account expected reduced pregnancy rates after a single three-day mating period following oestrus synchronization, a short natural mating period in the unsynchronized animals (often covering less than a full oestrus cycle) and year-round breeding, ewes were allowed a minimum of three mating attempts before they were classified as infertile as outlined above. Any ewes that were removed from the flock for unrelated reasons without being re-mated or before completing a minimum of three subsequent mating attempts were excluded from the analyses from that time point.

For lamb mortality parameters, the following definitions were used:

- *Abortion:* the spontaneous delivery of an immature, weak, or dead lamb prior to day 142 of gestation [20].
- *Mummification:* the delivery of a prematurely perished, dehydrated foetus at term.
- *Premature foetal death:* a mummified or aborted foetus.
- *Stillbirth:* the delivery of a dead, fully developed lamb in a fresh state at term.
- *Perinatal mortality:* any stillbirths plus postnatal lamb deaths up to the age of two days [21]

Parturition categories (mode of previous delivery) were classified as follows:

- *“only vaginal”:* vaginal delivery in an animal that is either primiparous or has previously only undergone vaginal deliveries.
- *“Caesarean section” (C section):* elective caesarean section performed in an animal that is either primiparous or has previously only undergone vaginal deliveries.
- *“Vaginal post C section”:* any follow-up vaginal deliveries in an animal that has previously undergone one elective caesarean section.

Statistical analyses were performed in R (version 4.3.1 (2023-06-16)). Uni- and multivariable Bayesian logistic regressions were applied for binary outcomes (conception, stillbirth, perinatal mortality, foetal death), and Bayesian linear regressions with the Gaussian distribution family were used for numeric outcomes (mating attempts, litter size, number of live-born lambs, birth weight). All analyses were performed via the “arm” package in R [22], which uses an approximate expectation-maximization algorithm to update the betas at each step using an augmented regression to represent the prior information and thus produces more reliable estimates for low-probability events (e.g., stillbirth or foetal death in our study) as compared to classic generalized linear models. The choice of linear models with Gaussian distribution was based on the findings published by MacDonald and White [23]. They demonstrated that data with small counts produce reliable estimates using linear regression with Gaussian distribution, as compared to Poisson distribution for counts or ordinal and multinomial models.

The inclusion of “individual ewe” as a random effect was attempted for all analyses but was not accepted for predictors linked to parturition records, since the individual ewes contributed very imbalanced numbers of repeated records to the analyses: 36.8% of the included animals were only represented by one paired parturition record that fell within the inclusion period, 33.3% of the ewes by two, and only 29.9% of the ewes by three or more paired records. Due to these imbalanced data, inclusion of “individual ewe” as a random effect did not improve the quality of the models for the assessment of subsequent conception, number of mating attempts, litter size, and number of live-born lambs, and was, therefore, rejected. Similarly, random effects were not used for the evaluation of stillbirth and foetal death due to very few positive cases for these outcomes, but were included in the models to assess influences on birth weight and perinatal mortality.

Age and parity were checked for multicollinearity and were found to contain different information, as many ewes did not lamb every year. Parturition records with missing values on any of the studied predictors were excluded from the analyses involving this particular predictor. From ewe-related data, the following response variables were studied: subsequent conception, the number of mating attempts required to achieve pregnancy, litter size, and the number of live-born lambs. For the evaluation of the two response variables “subsequent conception” and “number of mating attempts required to achieve pregnancy”, the predictors “season” and “reproductive management” could not be included, as any possible repeated mating attempts leading to pregnancy in a particular animal often took place at different times of year, and included different protocols in some. For these two response variables, the mode of previous delivery, ewe age, the number of previous parturitions (1, 2, or ≥3), and the presence of retained foetal membranes (yes/no) following the previous parturition were thus studied as predictors. For the evaluation of the potential effects on litter size and the number of live-born lambs, the following predictors were studied: mode of previous delivery, season of successful mating attempt (spring [March–May], summer [June–August], autumn [September–November], winter [December–February]), the reproductive management procedure leading to this pregnancy (synchronization with eCG, synchronization without eCG, not synchronized), retained placenta, ewe age, and parity. For the number of live-born lambs, the potential influence of litter size and the current mode of delivery leading to the birth of these lambs (vaginal delivery or caesarean section) were additionally considered in the analyses. Due to 28 missing values for retained placentas, this predictor could not be included in the multivariable models and was thus only assessed using univariable analyses for a reduced number of parturition records.

Response variables studied from lamb-related data included stillbirth, perinatal mortality, lamb birth weight, and premature foetal death. For the evaluation of stillbirth and perinatal mortality, the following predictors were studied: mode of previous delivery, ewe age, parity, litter size, lamb sex, lamb birth weight, and the current mode of delivery (vaginal delivery or caesarean section) leading to the birth of these lambs.

Lamb birth weight was studied using the mode of previous delivery, dam age, current parity (2 and ≥3), litter size, and lamb sex. For the assessment of premature foetal death, the mode of previous delivery, dam age, current parity and litter size were studied as predictors. Given the low number and high importance of all predictors, all models included all listed predictors as valuable confounders from the veterinary perspective. Therefore, no variable selection procedures were attempted. *p* < 0.05 was considered significant, while *p* < 0.1 was considered a tendency.

## 3. Results

### 3.1. Descriptive Results

Overall, sheep were re-bred following 423 of the 636 parturitions (66.5%) that took place in the studied period, leading to pregnancy on 95.0% of the occasions (402/423). The re-breeding percentage was 77.2% (233/302) following vaginal delivery, 78.2% (122/156) after the first elective caesarean section, 65.9% (56/85) after subsequent vaginal deliveries in ewes with a history of one elective caesarean section, and 11.3% (9/80) following a second caesarean operation. Three of these nine latter animals were retained for further parturitions thereafter. These figures do not include additional animals retained for breeding in the active flock, which were still awaiting subsequent breeding attempts or pregnancy outcomes by the end of the inclusion period (*n* = 52), since the eventual outcomes for these animals had not yet been determined.

On 141 occasions (22.2%), animals were removed from the flock without being subjected to any subsequent breeding attempts, while 19 animals (3.0%) were culled for reasons unrelated to fertility before completing a minimum of three mating attempts and were thus excluded from that time point. One animal was sold as pregnant, but no information of the subsequent pregnancy outcome was available; this pregnancy was, therefore, also excluded from the analyses. Twenty-one animals underwent a minimum of three subsequent mating attempts but did not conceive. These animals were thus classified as infertile. Reasons for removal from the flock (*n* = 182 removed animals) included completion of a second caesarean section (44.0% of removed animals), health issues unrelated to fertility such as, for instance, acute or chronic mastitis, dentition or injury, together accounting for 17.6% of the removed animals, age (animal-dependent assessment, no fixed age limit; 15.4%), breeding selection due to the production of lambs with unfavourable characteristics (5.5%), sale for breeding or as pets (4.9%), unrecorded causes (1.1%), and infertility in the above-mentioned 21 cases (11.5% of removed animals). Only one parturition-related ewe death occurred during the study period (1/636; 0.2%), involving a case of a retained placenta and subsequent metritis and peritonitis in a ewe 6 days after a second caesarean section. The uterine suture was intact in this animal [18].

Four hundred and eleven paired parturition and breeding records of 204 sheep could be evaluated for subsequent breeding outcomes following vaginal delivery (“only vaginal”, *n* = 233), elective caesarean section (“C section”, *n* = 122), and following subsequent vaginal deliveries in animals with a history of one prior elective caesarean operation (“vaginal post C section”, *n* = 56). A total of 695 lambs were born after 390 subsequent pregnancies, while mating attempts failed in 21 cases. Premature foetal death during subsequent pregnancy occurred in 24 lambs (2 aborted and 22 mummified foetuses), together accounting for 3.5% of all lambs born. The overall stillbirth and perinatal mortality rates (premature foetal deaths excluded) were 3.0% and 7.9%, respectively. The descriptive results of subsequent breeding success for the three previous delivery categories are summarized in Table 1, while the characteristics of any lambs born following subsequent pregnancies are shown in Table 2.

### 3.2. Results of Statistical Analyses

Retained placenta could not be included in the multivariable models due to a high number of missing values for this parameter, which would have led to the exclusion of the affected parturition records from the multivariable analyses. It was, however, assessed univariably for a reduced number of animals for which the relevant information was available. Dam age (*p* = 0.107) and parity (*p* = 0.673) were not significant for subsequent conception in univariable analyses, while the mode of previous delivery (*p* = 0.060) and the presence of retained foetal membranes at that time (*p* = 0.094) both showed a tendency. The subsequent multivariable model did not show any significance for either mode of previous delivery (*p* = 0.101), age (*p* = 0.248), or parity *p* = 0.869).

The mode of previous delivery (*p* = 0.015) and retained placenta (*p* = 0.010) showed significance in univariable analyses for the number of mating attempts necessary to achieve a subsequent pregnancy, while ewe age and parity were not significant. In the subsequent multivariable model, ewe age was, however, identified as most significant (*p* = 0.025), with an increased number of mating attempts in older ewes (estimate: 0.11; 95% CI 0.01–0.21; *p* = 0.026), but the mode of previous delivery also remained significant (*p* = 0.033). Parity showed a tendency (*p* = 0.081). Upon pairwise comparison amongst categories, there was a significantly decreased number of mating attempts following vaginal delivery compared to C section (estimate: −0.20; 95% CI −0.39–−0.02; *p* = 0.027), a significant reduction in mating attempts for the vaginal post C section category compared to C section (estimate: −0.29; 95% CI −0.58–−0.01; *p* = 0.041), and no significant difference between the two vaginal categories (*p* = 0.522). The detailed results of the pairwise comparisons for the multivariable model are shown in Table 3.

**Figure 1:**
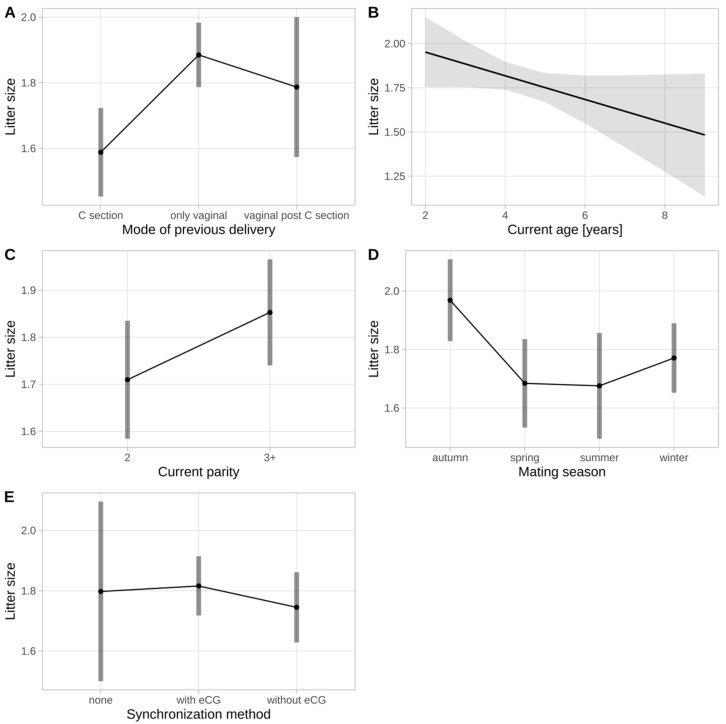
Predicted values of a multivariable Bayesian linear regression model to assess the influence of the mode of previous delivery (**A**), age (**B**), parity (**C**), mating season (**D**) and synchronization method (**E**) on litter size. The mode of previous delivery (*p* = 0.003) and the mating season (*p* = 0.021) showed significance, while ewe age showed a tendency to impact the results (*p* = 0.079). Error bars and shadows indicate the 95% confidence intervals. C section = caesarean section; eCG = equine chorionic gonadotropin.

Univariable analyses showed significance for the mode of previous delivery in relation to litter size (*p* = 0.003), with a reduced litter size for “C section” in comparison to the “only vaginal” category (estimate: −0.281; 95% CI −0.476–−0.086; *p* = 0.002). There was a tendency for age (*p* = 0.074) and mating season (*p* = 0.056), with smaller litters in older ewes, and larger litters when mating took place in autumn, while no significance was seen for parity, the method of synchronization or retained placenta. In the subsequent multivariable model, the mode of previous delivery remained significant (*p* = 0.003), while mating season also showed significance (*p* = 0.021), and age remained a tendency (*p* = 0.079). There were significantly larger litters in the “only vaginal” category as compared to “C section” (estimate: 0.30; 95% CI 0.13–0.47; *p* < 0.001), but there was no significant difference between “vaginal post C section” and “only vaginal”, or between “vaginal post C section” and “C section”. The results of these multivariable analyses are illustrated in Figure 1, and details are shown in Table 4.

The number of live-born lambs per ewe was highly correlated to the total number of lambs born per ewe (r = 0.83), with litter size (*p* < 0.001) over-riding all other predictors except for the mode of current delivery, which also showed significance (*p* = 0.030) in the multivariable model. There was a reduced number of live-born lambs for offspring born by vaginal delivery in comparison to lambs born by caesarean section (estimate: −0.09; 95% CI −0.17–−0.01; *p* = 0.031), but none of the other studied predictors (mode of previous delivery, ewe age, parity, mating season, and synchronization method) showed any significance in the multivariable model.

Only the mode of current delivery (univariable: *p* < 0.001; multivariable: *p* = 0.007) and lamb birth weight (uni- and multivariable: *p* < 0.001) showed significance for stillbirth in both the uni- and multivariable analyses (*n* = 639 lambs with full data sets). Neither the mode of previous delivery (univariable: *p* = 0.357; multivariable: *p* = 0.402) nor any of the other studied predictors (ewe age, parity, litter size, and lamb sex) showed any significance in this context. There was a lower probability of stillbirth for lambs with a higher birth weight (OR: 0.26; 95% CI 0.13–0.52; *p* < 0.001), and a higher probability of stillbirth for lambs born through vaginal delivery (OR: 15.6; 95% CI 0.99–246; *p* = 0.051) in comparison to caesarean section. Similarly, lamb birth weight was highly significant for perinatal mortality in the univariable analyses (*p* < 0.001), and the current mode of delivery also showed significance (0.018), with lower odds of survival for lighter lambs and lambs born through vaginal delivery. In the subsequent multivariable model (*n* = 639 lambs with full data sets), lamb birth weight retained its significance (*p* < 0.001), again with lower odds of perinatal mortality for heavier lambs (OR: 0.21; 95% CI 0,13–0.33; *p* < 0.001). Litter size also showed significance (*p* = 0.006), with lower odds of perinatal mortality for lambs from larger litters (OR: 0.46; 95% CI 0.25–0.87; *p* = 0.016), while lamb sex showed a tendency to have an impact (*p* = 0.053), with higher odds of perinatal mortality for male lambs (OR: 2.07; 95% CI 0.97–4.46; *p* = 0.062). 

In univariable analyses, litter size (*p* < 0.001) and lamb sex (*p* < 0.001) were highly significant for lamb birth weight, but the mode of previous delivery also showed significance (*p* = 0.002) in the univariable analysis, with higher birth weights in the C section group, in male lambs and in lambs from smaller litters. Parity showed a tendency to have an impact (*p* = 0.074), with heavier lambs born to ewes of higher parity. In the multivariable analyses (*n* = 642 lambs with full data sets), only litter size (*p* < 0.001) and lamb sex (*p* < 0.001) remained significant, with lower birth weights in larger litters (estimate: −0.69; 95% CI −0.79–−0.59; *p* < 0.001) and higher birth weights in male lambs (estimate: 0.30; 95% CI 0.19–0.42; *p* < 0.001).

For premature foetal death, the mode of previous delivery, ewe age and parity did not show any significance in univariable analyses, while litter size was significant (*p* < 0.001). The significance of litter size (*p* = 0.001) was confirmed using the multivariable model, with higher odds of premature foetal death for lambs from larger litters (OR: 2.61; 95% CI: 1.79–3.79; *p* < 0.001). Again, the multivariable model did not reveal any significance for any of the other three predictors.

## 4. Discussion

This study is the first to include future reproductive outcomes in sheep following vaginal delivery, elective caesarean section and, particularly, further post-caesarean vaginal deliveries, thus assessing the true long-term effects of caesarean section, and taking into account confounding factors for all analyses by applying multivariable models. The results of univariable analyses have been reported to aid comparison to other studies. Also, there was missing information on placental shedding for a number of parturitions—this predictor was, therefore, only studied univariably for a reduced number of parturition records with available information. When interpreting univariable analyses, it is important to recognize that they ignore the simultaneous effects of various potentially influential factors and are thus frequently confounded. For instance, ewes with a history of caesarean section had lambs with a significantly higher birth weight. They, however, also had significantly smaller litters, and only litter size (along with lamb sex) was identified as significant in the multivariable model. Without simultaneously considering litter size (and lamb sex) when assessing lamb birth weight, a false (positive) effect of caesarean sections on lamb birth weights might thus have been stated. Only the results of the multivariable models are, therefore, discussed here. In a previous study on the subject, confounders were only included in analyses studying lamb birth weight and weight gain, but not directly assessed for future reproductive performance such as conception, subsequent litter size, and the number of live-born lambs [7]. Our study fills this gap, and additionally allowed studying further follow-up pregnancies in animals with a history of an elective caesarean section to gain insight into the potential true long-term effects of the procedure.

In accordance with previous studies [7,15], we could not identify a significant effect of the mode of previous delivery on subsequent conception, and the observed conception rate of 91.0% immediately following a caesarean section, and of 94.6% following further vaginal deliveries in animals with a history of a caesarean section, was similar to the 89.4% reported in a Norwegian publication also studying elective procedures [7].

Ewe age was the most significant factor associated with an increased number of mating attempts to achieve subsequent pregnancy, but the mode of previous delivery also showed significance. The particular circumstances of the studied flock in relation to oestrus synchronization, year-round breeding, and very short natural mating periods, if applied, are likely to have made these effects come to light—it is, however, likely that these differences are not clinically relevant in field situations involving natural, seasonal breeding, and mating periods covering at least two full oestrus cycles. The clinical and economical significance of this observed effect is, therefore, debatable.

Neither stillbirth, perinatal mortality, premature foetal death, nor lamb birth weights were influenced by the mode of previous delivery, but an immediately preceding elective caesarean section was associated with a significantly reduced subsequent litter size, an effect also observed in a previous study from Norway [7]. However, ewes still produced an average of 1.6 lambs per parturition immediately following surgical delivery, and this reduction in litter size was no longer seen following further pregnancies in ewes with a history of a caesarean section, as shown by a lack of statistically significant differences between the “only vaginal” and “vaginal post C section” categories. The economic effect of this temporary reduction in litter size is, therefore, likely to be limited, particularly in younger ewes if they are retained for more than one subsequent pregnancy. 

It is, however, interesting to see this reduction in litter size despite the generally very long rest period in annually bred sheep, which is often cited to explain a lack of fertility problems in this species following certain events commonly adversely affecting fertility in cattle, such as, for instance, retained placenta [24] or uterine infection [25]. In the particular case of the studied flock, the inter-lambing interval even exceeded one year. While we were unable to directly assess post-partal uterine health or inflammation, it is possible that surgical intervention and subsequent healing may have triggered longer-standing intra-uterine inflammatory processes that might have affected subsequent litter size. Also, the occurrence of retained placenta was more frequent in surgical cases [18]. There has been little research interest in post-partal ovine uterine pathology, but clinical and subclinical endometritis, often caused by intrauterine contamination or retained placenta, have been extensively studied in cattle, and long-lasting negative effects on fertility have been described in this species, as recently reviewed by LeBlanc [26]. For instance, inflammation leads to the creation of unfavourable conditions for sperm survival, zygote development, implantation, and placentation, as well as negatively affecting ovarian and endocrine functions [27,28,29,30]. However, a study assessing a potential effect of experimentally induced intrauterine bacterial infection on subsequent fertility in sheep did not identify any negative effects on subsequent reproductive performance in the inoculated ewes [25]. Other studies in sheep showed that uterine involution was not negatively affected by intrauterine bacterial contamination [31] or experimentally induced metritis [32].

Dystocia, caesarean section, and retained placenta led to an initially slower uterine involution in the early post-partal stages in comparison to uneventful vaginal delivery in ewes in one study, but no ultrasonographic differences could be observed between the studied groups by day 30 post-partum [33]. Ovine uterine involution is generally considered complete by day 30 [33,34] or 34 post-partum [35]. A delayed histological regeneration in ewes with experimentally induced intrauterine infection in comparison to control animals was seen in another study [25], but no histological differences between the groups were observed by day 62 post-partum. Following a rest period of several months, as customary in annually bred ewes, uterine infections are generally expected to have cleared prior to re-mating [24,25]. Similar to cattle, subclinical endometritis can, however, persist for prolonged periods in individual ewes, as seen in the animals studied by Nasreldin et al. [36], who selected animals that repeatedly failed to conceive to characterize immunological, biochemical, and inflammatory properties of clinical and subclinical endometritis in sheep.

Piersanti et al. [37] showed persistent effects of metritis on bovine granulosa cell transcriptome even after the resolution of uterine disease, thus leading to potentially long-lasting effects on ovarian function. It is unclear if similar effects exist in sheep, and if post-surgical uterine healing processes or an increased incidence of retained foetal membranes after elective caesarean section [18] are sufficient to cause such an effect. If so, it may serve as an explanation for the observed reduction in litter size affecting the pregnancy immediately following a caesarean section. Obstruction of the fallopian tube due to salpingitis, which has been described as a sequel to metritis in sheep [7], or accidental ligation of the salpinx during surgery are also a theoretical possibilities—these effects are, however, expected to be permanent.

In cattle, uterine adhesions have been described following caesarean section and have been attributed to reduced fertility [38]. No adhesions or macroscopically obvious changes of the uterus were, however, seen in the 80 ewes operated on a second time in the presented study population. A more likely direct effect of surgery which may affect future fertility and pregnancy outcomes is uterine scar formation [39]. Localized scar tissue is likely to negatively affect implantation and placentation capacity of the affected area, thus potentially leading to a reduction in litter size. One study showed clear histological evidence of uterine scar tissue in examined sheep as determined by localized fibrosis eight months after caesarean surgery, and double-layer uterine closure led to a wider scar than a single-layer suture [40]. Our data confirm a reduction in litter size in the first pregnancy following caesarean delivery as also seen in one previous publication [7], but suggest that this reduced number of lambs born per ewe following caesarean section is limited to the first subsequent pregnancy. Time and further regenerative processes induced by the first post-caesarean pregnancy and parturition, therefore, seem to allow the ovine uterus to return to its pre-caesarean capacities. Immediate ewe and lamb survival rates following elective caesarean section have also been shown to be excellent [18]. While elective caesarean sections are not advocated for use in general practice, a better understanding of the largely absent direct effects of surgical delivery in sheep is important to encourage farmers to request this procedure more frequently for the treatment of severe dystocia. In practice, farmers often refrain from re-breeding ewes following caesarean section for fear of future fertility problems [1,5]. More importantly, welfare issues arise for ewes suffering from dystocia because many farmers are reluctant to ask for veterinary assistance [41,42]. The results of this study can, therefore, help to achieve better acceptance and wider use of emergency caesarean sections in sheep farming to ensure adequate treatment of dystocia.

## 5. Conclusions

A previous caesarean section did not significantly affect subsequent pregnancy rates or lamb mortality parameters in follow-up pregnancies. While there was a temporary reduction in litter size in the first post-caesarean pregnancy, litter size returned to pre-caesarean levels in further follow-up parturitions. This time-limited effect is, therefore, economically acceptable. The results fully encourage the use of caesarean sections in ovine obstetrics, and the observed excellent post-surgical reproductive outcomes support retention for breeding.

## Figures and Tables

**Table 1:** Descriptive results of ewe-related data: subsequent breeding success by mode of previous delivery. For pregnancy rate, *n* = 411 paired parturition and fertility records from 204 sheep; for number of mating attempts leading to pregnancy, litter size and number of live-born lambs: *n* = 390 subsequent pregnancies of 196 sheep (some sheep contributed more than one paired record to the analyses).

Mode of Previous Delivery	Only Vaginal	C Section	Vaginal Post C Section
**(*n* = 411 paired fertility records)** Subsequent pregnancy rate	**(*n* = 233)** 97.0% (226/233)	**(*n* = 122)** 91.0% (111/122)	**(*n* = 56)** 94.6% (53/56)
**(*n* = 390 pregnancy records)** Mean number of mating attempts to achieve subsequent pregnancy (median; range)	**(*n* = 226)** 1.4 (1; 1–5)	**(*n* = 111)** 1.6 (1; 1–5)	**(*n* = 53)** 1.8 (2; 1–7)
Mean subsequent litter size (median; range)	1.9 (2; 1–4)	1.6 (2; 1–4)	1.8 (2; 1–7)
Mean subsequent number of live-born lambs (median; range)	1.8 (2; 0–4)	1.5 (1; 0–4)	1.6 (2; 1–3)

Only vaginal: natural delivery in an animal that was either primiparous or has previously only undergone vaginal deliveries; C section: elective caesarean section on immediately preceding occasion in an animal that was either primiparous or has previously only undergone vaginal deliveries; vaginal post C section: any further vaginal deliveries in an animal with a history of one elective caesarean section.

**Table 2:** Descriptive results of lamb-related data: viability and birth weights of 695 lambs born following 390 pregnancies of 196 sheep by mode of previous delivery. (Some sheep contributed more than one paired parturition record to the analyses).

Mode of Previous Delivery	Only Vaginal (*n* = 226)	C Section (*n* = 111)	Vaginal Post C Section (*n* = 53)
Number of lambs born at subsequent parturition	426	176	93
Premature foetal death in subsequent pregnancy	Aborted: *n* = 1 (0.2%) Mummified: *n* = 10 (2.3%)	Aborted: *n* = 1 (0.6%) Mummified: *n* = 6 (3.4%)	Aborted: *n* = 0 (0%) Mummified: *n* = 6 (6.5%)
Stillbirth rate at subsequent parturition	3.6% (15/415)	2.4% (4/169)	1.1% (1/87)
Perinatal mortality rate at subsequent parturition	8.0% (33/415)	8.3% (14/169)	6.9% (6/87)
Mean birth weight [kg] at subsequent parturition (median; range); *n* = number of lambs with available information	3.90 (3.9; 0.4–7.2) (*n* = 403)	4.3 (4.3; 1.5–6.7) (*n* = 160)	4.2 (4.2; 1.5–6.8) (*n* = 86)

Only vaginal: natural delivery in an animal that was either primiparous or has previously only undergone vaginal deliveries; C section: elective caesarean section on immediately preceding occasion in an animal that was either primiparous or has previously only undergone vaginal deliveries; vaginal post C section: any further vaginal deliveries in an animal with a history of one elective caesarean section.

**Table 3:** Pairwise comparisons of predictors for the number of subsequent mating attempts to achieve pregnancy following a multivariable Bayesian linear regression model to include mode of previous delivery, ewe age, and parity; *n* = 390 paired parturition records.

Predictor	Estimate	95% CI	*p*-Value
***Previous parturition***			
Only vaginal–C section	−0.20	−0.39–−0.02	0.027
Vaginal post C section–C section	−0.29	−0.58–−0.01	0.041
Vaginal post C section–only vaginal	−0.09	−0.37–0.19	0.522
***Previous age***	0.11	0.01–0.21	0.026
***Previous parity***			
2-1	−0.14	−0.36–0.07	0.186
(3+)-1	−0.37	−0.70–−0.04	0.026
(3+)-2	−0.23	−0.48–0.03	0.083

CI = confidence interval; only vaginal: natural delivery in an animal that is either primiparous or has previously only undergone vaginal deliveries; C section: elective caesarean section on immediately preceding occasion in an animal that was either primiparous or has previously only undergone vaginal deliveries; vaginal post C section: any further vaginal deliveries in an animal with a history of one elective caesarean section.

**Table 4:** Pairwise comparisons of predictors for litter size following a multivariable Bayesian linear regression model to include the mode of previous delivery, mating season, method of synchronization, ewe age, and parity; *n* = 390 paired parturition records.

Predictor	Estimate	95% CI	*p*-Value
***Previous parturition***			
Only vaginal–C section	−0.30	0.13–0.47	<0.001
Vaginal post C section–C section	0.20	−0.05–0.44	0.112
Vaginal post C section–only vaginal	−0.10	−0.34–0.15	0.436
***Current age***	−0.07	−0.14–0.01	0.080
***Current parity***			
(3+)-2	0.14	−0.05–0.33	0.142
***Mating season***			
Spring–autumn	−0.28	−0.49–−0.08	0.007
Summer–autumn	−0.29	−0.52–−0.06	0.013
Summer–spring	−0.01	−0.24–0.23	0.943
Winter–autumn	−0.20	−0.38–−0.01	0.034
Winter–spring	0.09	−0.11–0.28	0.383
Winter–summer	0.10	−0.12–0.31	0.390
***Synchronization method***			
With eCG–none	0.02	−0.30–0.33	0.909
Without eCG–none	−0.05	−0.37–0.27	0.746
Without eCG–with eCG	−0.07	−0.23–0.09	0.374

CI = confidence interval; eCG = equine chorionic gonadotropin; only vaginal: natural delivery in an animal that is either primiparous or has previously only undergone vaginal deliveries; C section: elective caesarean section on immediately preceding occasion in an animal that is either primiparous or has previously only undergone vaginal deliveries; vaginal post C section: any vaginal deliveries in an animal with a history of one elective caesarean section.

## Data Availability

The data presented in this study are available on request from the corresponding author. The data are not publicly available due to institutional policies.
